# URGENCY HOSPITALIZATIONS FOR INGUINAL HERNIA IN BRAZIL FROM 2010 TO 2019: MORTALITY AND COSTS IN THE PUBLIC HEALTH SYSTEM

**DOI:** 10.1590/0102-672020230020e1738

**Published:** 2023-07-07

**Authors:** Danilo Oliveira Amaral, Jonatan Eduardo Silva, Lenise Moreira da Silva, Frank Luiz Pereira Carnesi, Felix André Sanches Penhavel, Renato Miranda de Melo

**Affiliations:** 1 Universidade Federal de Goiás, Faculty of Medicine – Goiânia (GO), Brazil; 2 Universidade Católica de Brasília, Faculty of Medicine – Brasília (DF), Brazil; 3 Universidade Federal de Goiás, Department of Surgery – Goiânia (GO), Brazil.

**Keywords:** Hernia, inguinal, Emergencies, Hospitalization, Mortality, Costs and cost analysis, Hérnia inguinal, Emergências, Hospitalização, Mortalidade, Custos e análise de custo

## Abstract

**BACKGROUND:**

It is known that elective inguinal hernioplasties are safe procedures, but in an emergency setting, they have higher rates of complications and hospital costs. Despite this, quantitative studies on the subject in Brazil are still scarce.

**AIMS:**

To evaluate the trend in hospitalization rates, hospital mortality, and costs for inguinal hernia in an emergency, regarding gender and age group.

**METHODS:**

This is a time series study with data from the Unified Health System (SUS), at the national level, from 2010 to 2019.

**RESULTS:**

The overall hospitalization rate (p=0.007; b<0,02) in all age groups (p<0.005; b<0) in both genders indicated a decreasing trend. The general mortality rate in both genders and in most age groups showed an increasing trend (p<0.005), as well as the cost of hospitalization in all age groups of both genders.

**CONCLUSIONS:**

Urgent hospitalization rates for inguinal hernia in Brazil have shown a steady or decreasing trend; however, hospital mortality and costs per hospitalization have demonstrated an increasing trend in recent years.

## RESUMO

**RACIONAL:**

Sabe-se que as hernioplastias inguinais eletivas são procedimentos seguros, mas que, em caráter de urgência, têm maiores taxas de complicações e custos hospitalares. Apesar disso, ainda são escassos os estudos quantitativos sobre o tema no Brasil.

**OBJETIVOS:**

Avaliar a tendência das taxas de internação, mortalidade hospitalar e custos por hérnia inguinal em caráter de urgência, quanto ao sexo e faixa etária.

**MÉTODOS:**

Trata-se de um estudo de séries temporais com dados do Sistema Único de Saúde (SUS), em nível nacional, no período de 2010 a 2019.

**RESULTADOS:**

A taxa de internação geral (p=0,007; b<0,02) em todas as faixas etárias (p<0,005; b<0) nos dois sexos indicou tendência decrescente. A taxa de mortalidade geral em ambos os sexos e na maioria das faixas etárias apresentou tendência crescente (p<0,005), assim como o custo da internação em todas as faixas etárias de ambos os sexos.

**CONCLUSÕES:**

As taxas de internação em caráter de urgência por hérnia inguinal, no Brasil, apresentaram tendência estacionária ou decrescente, entretanto, a mortalidade hospitalar e os custos por internação mostraram tendência crescente nos últimos anos.

## INTRODUCTION

It is known that 5% of the population will develop an abdominal wall hernia, approximately 75% in the inguinal region, two thirds indirect hernias and the remaining direct. Men are 25 times more likely to present hernias than women and the prevalence increases with age; it occurs more commonly on the right side^1,6,10^.

Most herniorrhaphies are performed electively, but urgent operations are necessary for the treatment of strangulated inguinal hernias or those with other complications^9,17^. Under these circumstances, studies carried out in the United States showed that patients undergoing urgent herniorrhaphy have higher mortality and readmission rates, in addition to higher hospital costs^11,14,19^. Despite the relevance of the topic, there are few studies that comprehensively assess the epidemiological behavior of these cases.

In Brazil, the Unified Health System (SUS) uses the Hospital Information System (HIS/SUS) to register the payment of services provided by hospital units (public or private) based on its own table. Considering that clinical outcomes and their economic impact are worse in emergencies, epidemiological studies may provide subsidies for the formulation of more appropriate public policies in the treatment of this condition. The aim of this study was to evaluate, over a decade, the trend in these rates, broken down by gender and age group.

## METHODS

This is a descriptive study with a quantitative approach of time series on hospitalization and hospital mortality rates and costs for inguinal hernia treated on an emergency basis, in Brazil, from January 2010 to December 2019.

Data from the Hospital Information System of the Department of Informatics of the Unified Health System (HIS/DATASUS) and population data from the estimates of the Interagency Health Information Network (IHIN) were used. In the admission records, based on the Authorization for Hospital Admission (AHA) forms, there are no specifications of the hernia disease, such as uni/bilaterality or crural/femoral location. Epidemiological variables such as gender, age group (AG), hospitalization rate, hospital mortality rate and hospitalization costs were considered. Four AGs were defined for this study: up to 19 years, 20 to 39 years, 40 to 59 years, and 60 years or older.

Data were tabulated using the TabWin (DATASUS) program and calculations were performed using the Microsoft Excel^®^ program. For the analysis of temporal trends, the Prais-Winsten regression method was applied. The logarithmized operational and morbidity indicators corresponded to the model-dependent variables, and the year, to the independent variable. The equation used for the regression model was as follows:

log(Yt)=ß0+ß1x(log(Yt)=logarithmized values of the dependent variable; ß0=constant or intercept; ß1 = linear trend coefficient; x=residual term)

There was no need for the Human Research Ethics Committee of the Federal University of Goiás (UFG) to approve the project, as it is a study with secondary data without the participants’ identification.

## RESULTS

**Figure 1:**
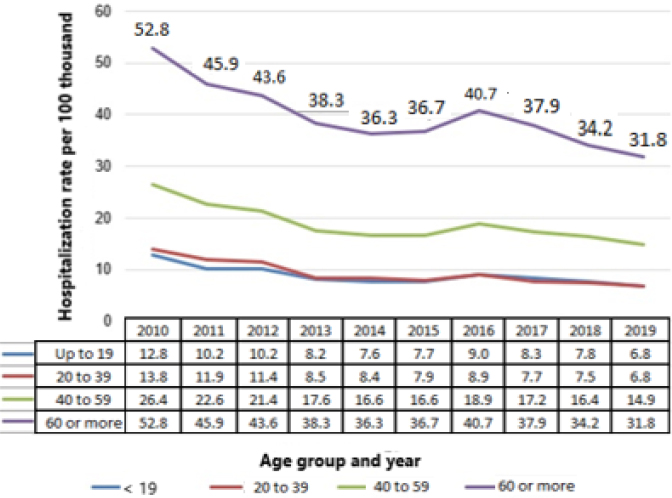
Average rates of hospitalization for inguinal hernia per 100,000 inhabitants in Brazil between 2010 and 2019.

A total of 302,032 emergency hospitalizations for inguinal hernias were analyzed from 2010 to 2019, of which 250,788 were male and 51,244 were female (Figure 1). The average overall hospitalization rate was 14.89 hospitalizations/100,000 inhabitants, a variable that indicated a decreasing trend in the period (p=0.007; b≤0.02), both for males (p=0.011; b≤0.018) and females (p=0.001; b≤0.029).

The highest average hospitalization rate in the period was observed in the AG 60 years or older (Figure 1). However, all AGs showed a decreasing trend in the period: up to 19 years (p=0.007; b≤0.024), 20 to 39 years (p=0.001; b≤0.030), 40 to 59 years (p=0.04; b≤0.025), and 60 years or older (p=0.002; b≤0.021).

In the same period, 1,772 deaths were recorded, of which 1,209 were male and 563 were female.

The mean hospital mortality rate for males was 0.49 deaths/100,000 admissions and for females it was 1.14 deaths/100,000 admissions. The temporal trend of hospital mortality rates was increasing, both overall (p=0.0001; b≥0.035) and by gender – male (p=0.0001; b≥0.037) and female (p=0.0001; b≥0.0359). The death rate recorded in women was more than twice that of men.

**Figure 2:**
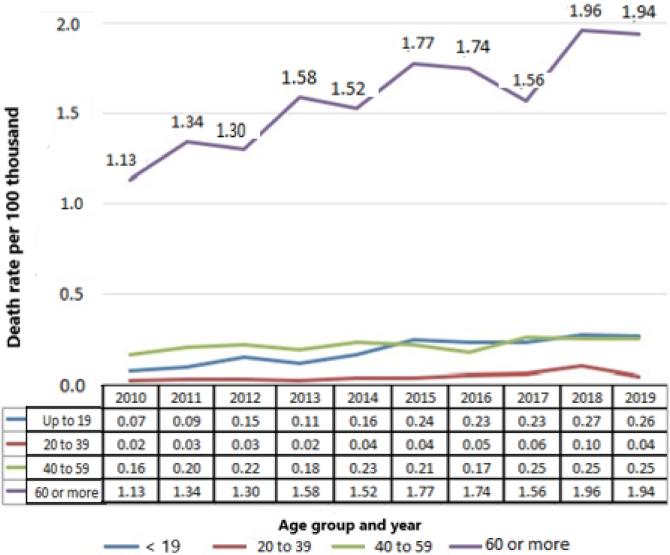
Average mortality rates due to inguinal hernia per 100,000 inhabitants in Brazil between 2010 and 2019.

The highest average hospital mortality rates were found in the AG 60 years or older. The temporal trend in the AG 40 to 59 years (p=0.351; b≤0.016) remained stationary, but all the others showed an increasing trend: up to 19 years (p=0.0001; b≥0.064), from 20 to 39 years (p=0.0001; b≥0.063) and 60 years or older (p=0.0001; b≥0.0229) (Figure 2).

Regarding the costs, R$ 175,960,550.60 were spent in Brazil during the period studied. The general average cost per hospitalization was R$ 587.01: R$ 581.06 spent for males and R$ 618.09 for females. The overall cost per hospitalization showed an increasing trend (p=0.0001; b≥0.012), similar for males (p=0.0001; b≥0.011) and females (p=0.0001; b≥0.016).

**Figure 3:**
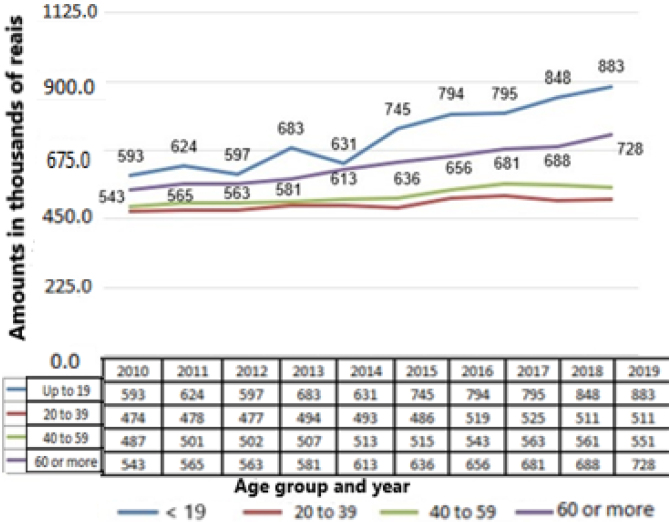
Average hospitalization costs in thousands of reais, per inguinal hernia, in Brazil between 2010 and 2019.

The hospital costs that had the highest mean values were those of the AGs extremes, which were up to 19 years and 60 years and older (Figure 3), with increasing trends in all AG: up to 19 years (p=0.0001; b≥0.020), 20 to 39 years (p=0.001; b≥0.004), 40 to 59 years (p=0.0001; b≥0.006) and 60 years or older (p=0.0001; b≥0.014).

## DISCUSSION

The present study used secondary data on hospitalizations for urgently operated inguinal hernias and identified a higher absolute number of hospitalizations and deaths in the male population. These findings are in agreement with another study carried out by Takahashi et al.^18^, who assessed the Brazilian population over a period of two years, also with secondary data on inguinal hernias treated on an elective or urgent basis and showed an inverse pattern in relation to femoral hernias. This was also demonstrated by another Brazilian study by Coelho et al.^5^, in which 88.7% of the population assessed with this condition was female, in a ratio of 8:1 in relation to males. A study conducted by Funes et al.^7^ with 51 patients with bilateral inguinal hernia reported that the disease is more frequent in men over 50 years old, who had approximately a 15 to 25% chance of developing the disease during their lifetime. This fact was attributed to the work activity, in this case, of an eminently manual nature.

Studies performed by Koch et al.^8^ and Nilsson et al.^12^ regarding treatments performed on urgent basis, with data from the Swedish Hernia Registry, demonstrated a higher number of hospitalizations in females, contrasting with the results of this research. Therefore, it is very likely that the diagnosis and elective treatment of inguinal hernias in the female population are performed earlier, thus avoiding the need for emergency surgery.

When analyzing the proportion of hospitalizations due to AG, it is observed that they increase with age. These observations are in agreement with a retrospective study carried out in districts of Oxford (England) by Primatesta et al.^14^, with data from elective and emergency admissions, involving more than 30,000 patients. The authors recorded higher rates of hospitalization, on an emergency basis, in patients with more advanced age, especially over 65 years old. Furthermore, a study on operative mortality due to inguinal hernia by Nilsson et al.^13^ in Sweden, demonstrated that the mean age in emergency surgeries was approximately 10 years higher than in elective repairs (70 vs 57.8 years).

It is recognized that the elective repair of inguinal hernias, mainly in a minimally invasive way, is low risk, has low mortality, lower costs, and the possibility of local anesthesia; nevertheless, it is still neglected by most surgeons, as shown in the Brazilian study written by Claus et al.^3^. In emergency operations, however, this risk increases. The present study identified low mortality rates in Brazil, although with an increasing trend in most of the AG evaluated. A study performed by Shyam et al.^15^, showed that this fact warns us of the convenience of treating patients on an elective basis, avoiding emergency surgeries and their possible complications, which was also verified concerning femoral hernia in the population analyzed by Coelho et al.^5^. In the Swedish Hernia Registry by Nilsson et al.^13^, mortality after elective and urgent inguinal hernia surgery was compared over a period of 13 years (1992 to 2005), and the result was seven times higher in the emergency setting. Furthermore, the authors concluded that mortality was twenty times higher when intestinal resection was necessary.

Female mortality in our study was significant and further research is needed to explain this finding. Information from the Swedish Hernia Registry, between 1992 and 2003, identified that the proportion of enterectomies in women was higher than in men, 16.6% and 5.6%, respectively. Femoral hernias were registered in 41.6% of women, compared to only 4.6% of men, which could explain the higher mortality found in emergency surgeries in women in studies carried out by Claus et al.^4^, and Koch et al.^8^. It is important to emphasize that the database used in the present study did not distinguish femoral hernias, which are more frequent in women.

Analyzing the deaths, it is also fundamental to consider the AG. In Brazil, all AGs showed an increasing trend, except for those aged 40 to 59 years (stationary). Although the percentages are small, these trends also point to the need to assess the quality of health care provided, which could be mitigated by public policies aimed at this disease. Perhaps it is better to address the condition before it becomes a surgical urgency or emergency.

The information obtained in this study corroborate the literatures, once the highest mortality rates were found at more advanced ages. The justification is the greater difficulty in establishing the diagnosis of an emergency surgical condition, either due to the presence of vague symptoms or even the higher prevalence of comorbidities in this population, which increase the probability of cardiovascular and pulmonary complications, among others. Studies produced by Nilsson et al.^12,13^ demonstrated that of the patients who died as a result of urgent surgical treatment, 47% had cardiovascular complications and 21% had pulmonary complications. Furthermore, a survey conducted by Primatesta et al.^14^, demonstrated that such information may be underestimated, since hernia is not always placed as the cause of death.

As for the cost per hospital stay, the trend was also growing. A study carried out in a general hospital in southern Brazil by Silva et al.^16^, demonstrated that herniorrhaphy in an elective outpatient regimen represented an average saving of 26.6% compared to patients hospitalized in an emergency setting. It also presented important benefits for the SUS and the patients themselves, such as shorter hospital stays, higher bed turnover, higher occupancy rates, and consequently, a reduction in the waiting list for other more complex operations.

Considering the distribution of hospital costs, higher mean values were found in AGs extremes, which were up to 19 years and 60 years or older. It is justifiable that the latter had the highest average cost per hospitalization, especially if we consider the higher frequency of comorbidities in this group. In contrast, a study conducted by Verhelst et al.^19^ demonstrated higher hospital costs due to inguinal hernia admissions in young people, notably in a cohort with a premature infant population, which revealed significantly higher costs in emergency surgeries (about 60%) compared to elective surgeries.

It is important to emphasize that the present study used secondary data from the HIS/SUS for the analysis of hospital costs. Therefore, the numbers and values of hospitalizations may be underestimated, since they are based only on the amount reimbursed by the SUS, and not on the actual cost of each hospitalization. Furthermore, as presented in the study by Bittencourt et al.^2^, accumulated inflation over the survey period (one decade) should be considered, which may have further contributed to this lag.

## CONCLUSIONS

Urgent hospitalization rates for inguinal hernia in Brazil have shown a steady or decreasing trend; however, hospital mortality and costs per hospitalization have demonstrated an increasing trend in recent years. Although the methodology used does not allow for establishing a direct causality relationship, it may provide subsidies for the elaboration of more realist public policies in health care. New studies are necessary to evaluate different associations between these trends and confront them with elective treatment, contributing to the improvement of care and cost adequacy in the treatment of inguinal hernia disease.
